# Lignocellulose integration to 1G-ethanol process using filamentous fungi: fermentation prospects of edible strain of *Neurospora intermedia*

**DOI:** 10.1186/s12896-018-0444-z

**Published:** 2018-08-17

**Authors:** Ramkumar B. Nair, Osagie A. Osadolor, Vamsi K. Ravula, Patrik R. Lennartsson, Mohammad J. Taherzadeh

**Affiliations:** 1 Swedish Centre for Resource Recovery, University of Borås, 50190 Borås, SE Sweden; 2 Mycorena AB, Stena Center 1 A, 41292 Gothenburg, SE Sweden

**Keywords:** Integration, Lignocelluloses, Bioethanol, Edible filamentous fungi, *Neurospora intermedia*

## Abstract

**Background:**

Integration of first- and second-generation ethanol processes is one among the alternate approaches that efficiently address the current socio-economic issues of the bioethanol sector. Edible filamentous fungus capable of utilizing pentoses from lignocelluloses and also possessing biomass application as potential animal feed component was used as the fermentation strain for the integration model. This study presents various fermentation aspects of using edible filamentous fungi in the integrated first and second generation ethanol process model.

**Results:**

Fermentation of edible strain of *N. intermedia* on the integrated first and second-generation ethanol substrate (the mixture of dilute acid pretreated and enzymatically hydrolyzed wheat straw and thin stillage from the first-generation ethanol process), showed an ethanol yield maximum of 0.23 ± 0.05 g/g dry substrate. The growth of fungal pellets in presence of fermentation inhibitors (such as acetic acid, HMF and furfural) resulted in about 11 to 45% increase in ethanol production as compared to filamentous forms, at similar growth conditions in the liquid straw hydrolysate. Fungal cultivations in the airlift reactor showed strong correlation with media viscosity, reaching a maximum of 209.8 ± 3.7 cP and resulting in 18.2 ± 1.3 g/L biomass during the growth phase of fungal pellets.

**Conclusion:**

*N. intermedia* fermentation showed high sensitivity to the dilute acid lignocellulose pretreatment process, with improved fermentation performance at milder acidic concentrations. The rheological examinations showed media viscosity to be the most critical factor influencing the oxygen transfer rate during the *N. intermedia* fermentation process. Mycelial pellet morphology showed better fermentation efficiency and high tolerance towards fermentation inhibitors.

## Background

The second generation (2G) lignocellulose-to-ethanol process that intends to reduce the dependence of first generation (1G) ethanol on the food grains, has gained much attention by researchers and industry, over the past few decades [1]. However, the existing 1G-ethanol processes continue to be favored over the 2G processes due to the surplus production of grains (wheat or corn), especially in Europe and the USA, together with the associated technical and economic challenges of 2G processes. A smart alternative approach to address this issue is to develop a model *‘integrating both first- and second-generation ethanol processes*’. The model first described by Lennartsson et al. [2] especially for the grain-based first- and second-generation ethanol processes, proposed the use of grain-derived lignocellulose waste such as straw, stover, bran, or stillage (waste stream from the 1G-ethanol process), as the 2G-ethanol substrate. The integration model hence opens-up a new avenue for converting the existing 1G-ethanol plants to a lignocellulose based *‘biorefinery’*, utilizing the existing infrastructure facilities such as distillation column, reactors, evaporators, etc. [3, 4]. This could also possibly cut down the investment cost and the associated risks that are currently faced by the stand-alone 2G lignocellulose-to-ethanol plants. The ease of lignocellulose availability at the vicinity of the 1G plant also creates an advantage towards the collection and transportation (logistics) system, which otherwise is a challenging issue faced by the 2G-ethanol process [4–6].

It is estimated that in 2017 nearly 202, 383 and 71 first generation (sugar/ starch based) ethanol refineries with a total production capacity (of the facilities) of about 59.3, 39.6 and 8.5 billion liters per year, currently exist in the USA, Brazil, and Europe, respectively [7]. Hence, introducing the lignocellulose integration model at these 1G-ethanol refineries could successfully render a technically and economically sustainable 2G-ethanol process [8–10]. However, the use of 1G-ethanol fermenting microorganisms, such as *Saccharomyces cerevisiae* (yeast), hinders the fermentation of pentose sugar from the lignocellulose biomass. It should be considered that the use of genetically modified pentose consumers can create socio-regulatory issues as well as it affects the quality of the animal feed product, DDGS (distiller’s dried grains with solubles) that contributes to a major share of the economics at the 1G-ethanol facility [11]. Therefore, finding the right microorganism for ethanol fermentation, capable of consuming pentose sugars and simultaneously maintaining the quality of DDGS narrows the options down to using the edible strains of filamentous fungi as the fermentation microbe [2].

The choice of filamentous fungi as a key player in *‘ethanol biorefinery’* has been initiated almost 15 years ago, when *Rhizopus oryzae* was used to produce ethanol from paper pulp sulfite liquor (a waste stream from pulp and paper industry) [12]. Since then several research and pilot scale studies have been carried out to explore the ethanol fermenting potential of various strains of edible filamentous fungi [13–15]. Many filamentous fungal strains are considered as GRAS *(generally regarded as safe)* microorganisms under Sections 201(s) and 409 of the Federal Food, Drug, and Cosmetic Act of the Food and Drug Administration (US-FDA) and also meet the requirements of being in the list of *Microbial food cultures* (MFC) of the EU regulation described under the *Qualified Presumption of Safety* (QPS) protocol introduced by the European Food Safety Authority (EFSA) [16]. This could be an important aspect should the fungal biomass obtained from the integration model be used either as an animal feed component or enrich the DDGS quality. However, the use of edible strains of filamentous fungi as the fermenting microbe in an integrated first and second-generation ethanol process model has not been studied previously. Hence, a deeper understanding of the process conditions such as the media fluid-rheology, fungal growth pattern and the effect of fermentation inhibitors, needs to be investigated and optimized before the desired outcome of the integration process model can be achieved.

In this study, *Neurospora intermedia* an edible strain of filamentous fungi was hence used as a model organism for the filamentous fungi based lignocellulose (wheat straw) integration to the existing first-generation wheat grain-to-ethanol process. The effect of lignocellulose pretreatment conditions, together with the inhibitor’s effect, on the fungal fermentation was determined. The morphological and rheological aspects were also investigated for an optimized ethanol and biomass production in the integrated first and second-generation ethanol process model.

## Methods

### Fungal strain

*Neurospora intermedia* CBS 131.92 (Centraalbureau voor Schimmelcultures, The Netherlands), an edible filamentous ascomycete, was used as the model-fermentation microorganism in the present study. The fungal strain was maintained and the inoculum preparation was followed as previously described [17]. Inoculum in the form of (a) fungal spores, 3–5 mL spore suspension (per L medium) with a spore concentration of 5.7 ± 1.8 × 10^5^ spores/ml; (b) mycelial filamentous biomass (0.2 to 0.3 g/L wet weight content) and (c) fungal pellets (0.1 to 0.8 g/L wet weight content), obtained following the methods specified by Nair et al. [17], were used throughout the cultivations.

### Substrate

Wheat straw (92.4% dry content) used in the demonstration scale pretreatment experiments and thin stillage (a residual product from the wheat based first generation ethanol facility) used for the integration experiments were supplied by Lantmännen Agroetanol (Norrköping, Sweden). Straw, with the composition (g/g, dry basis) arabinan 0.048 ± 0.013; galactan 0.0053 ± 0.0015; glucan 0.315 ± 0.061; mannan 0.0047 ± 0.0011; and xylan 0.24 ± 0.08, was milled (0.2–0.25 mm size) using a rotor beater mill before use. Thin stillage with a natural pH of 3.5 was characterized with the composition of total solids (% *w*/*v*) 9.2 ± 0.4 and suspended solids (% w/v) 2.2 ± 0.6 and (g/L) total nitrogen 4.8 ± 0.5; xylose 0.8 ± 0.1; arabinose 1.5 ± 0.1; glycerol 7.0 ± 0.; lactic acid 1.8 ± 0.1; acetic acid 0.21 ± 0.01 and ethanol 1.2 ± 0.2.

### Pretreatment and hydrolysis: Preparing the fermentation substrate

The dilute-phosphoric acid pretreatment of wheat straw was carried out in a 30-L one-step vertical plug-flow continuous reactor at a Biorefinery Demo Plant (RISE, Örnsköldsvik, Sweden). Based on the preliminary laboratory study [18], three pretreaments were carried out at the demonstration plant at different conditions (Table 1). Based on the pretreatment temperature, the straw pre-hydrolysates obtained from the demo-plant, were designed as P_201_, P_195,_ and P_190_. The chemical characteristics of the pretreated slurry are depicted in Table 1. Pretreatment was carried out as explained in a previous study [18]. The pretreated straw slurry P_201,_ P_195,_ and P_190_ (with slurry pH, 3.5 2.9 and 3.2 respectively) was subjected to enzymatic hydrolysis without any pre-washing at solid loading 3.5, and 7.0%, at pH 5.0 ± 0.3 (adjusted with 2 M NaOH) at 50.0 ± 0.2 °C water bath at an enzyme loading of 10 FPU/g substrate dry weight. Cellulase enzyme Cellic CTec2 (Novozymes, Denmark) with 134 FPU /mL activity was used for the hydrolysis. The hydrolysate obtained after the enzyme hydrolysis of pretreated slurry P_201,_ P_195,_ and P_190_ were designated as hydrolysate H_201,_ H_195,_ and H_190,_ respectively.

**Table 1:** Characteristics of the dilute-phosphoric acid pretreated wheat straw pre-hydrolysate from the demonstration facility

*Characteristics*		Pretreatment conditions		
		P_201_	P_195_	P_190_
Acid concentration		0.7% (w/v)	1.2% (*w*/*v*)	1.75%
Residence time		7 min	7 min	10 min
Temperature		201 ± 4 °C;	195 ± 2 °C,	190 ± 2 °C
Composition of pretreated slurry				
Sugars in liquid (g /L) and solid (g/g dry substrate) fraction				
Arabinan	Liquid	5.04 ± 0.1	6.6 ± 0.2	15.02 ± 0.14
	Solid	0.031 ± 0.003	0.028 ± 0.001	0.022 ± 0.002
Glucan	Liquid	2.8 ± 0.2	4.6 ± 0.4	11.53 ± 0.06
	Solid	0.302 ± 0.010	0.246 ± 0.02	0.235 ± 0.01
Xylan	Liquid	15.8 ± 0.4	31.6 ± 0.1	16.1 ± 0.1
	Solid	0.016 ± 0.003	0.028 ± 0.007	0.037 ± 0.006
Fermentation Inhibitors (g /L) in liquid fraction				
Acetic acid		2.1 ± 0.0	2.8 ± 0.1	1.82 ± 0.03
Furfural		3.2 ± 0.3	5.6 ± 0.4	2.71 ± 0.12
HMF		0.39 ± 0.02	0.53 ± 0.02	0.89 ± 0.03

### Neurospora intermedia fermentation for the integration process

**Fig. 1:**
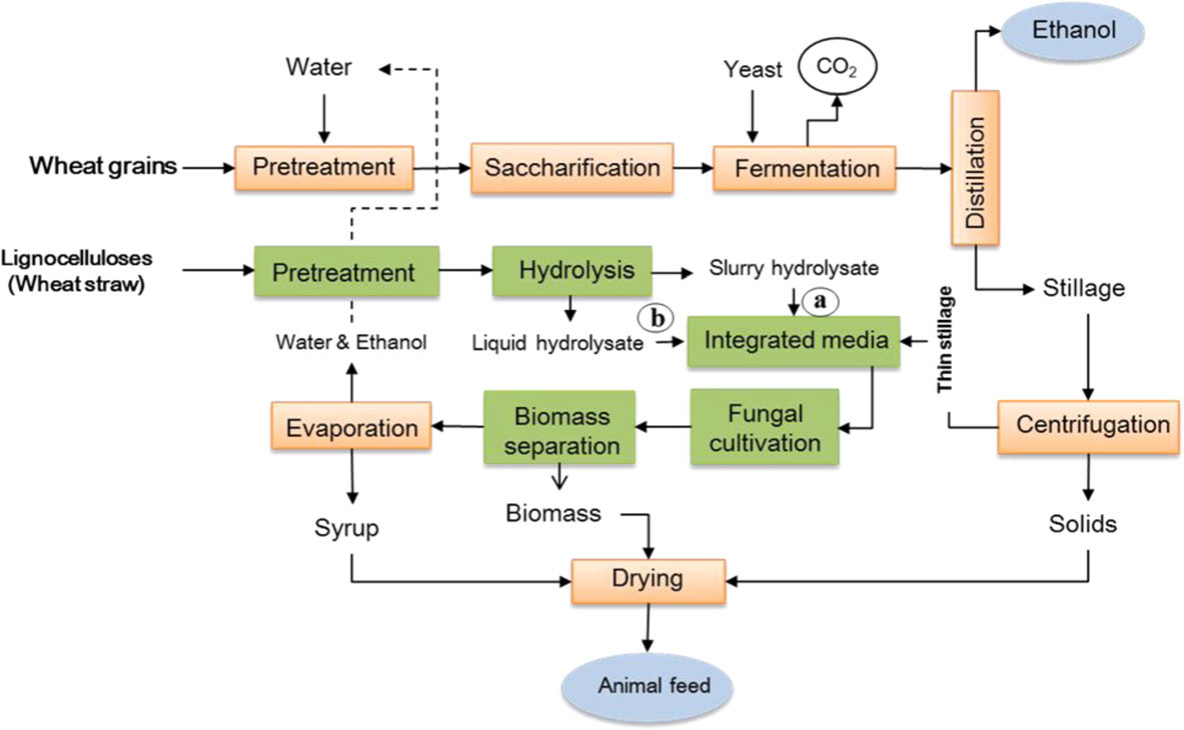
Integration model for the first and second generation bioethanol process at the existing wheat based ethanol facilities using edible filamentous fungus, *N. intermedia.* Fermentation media for the integration model was developed using a) whole lignocellulosic slurry or b) liquid part of the slurry hydrolysate (*modified from* [2]*)*

The general schematic for the integrated first and second-generation ethanol process is shown in Fig. 1. The hydrolyzed wheat straw H_190,_ H_195,_ and H_201_ at different solid loading concentrations (*w*/*v*) of 3.5, and 7.0%, were used for the fungal fermentation. The hydrolysates, either in the form of slurry (solid and liquid) or as liquid supernatant (obtained after centrifugation of the hydrolysate- slurry at 15,000 *g*), were mixed with thin stillage (total solids 8%) at ratio 1:1 to form the fermentation media. Cultivations were made in 250 ml Erlenmeyer flasks (100 ml liquid volume) at 35 °C and 150 rpm in an orbital shaking water bath (Grant OLS-Aqua pro, UK), for 120 h. Control fermentation experiments were carried out separately in straw hydrolysates (both liquid and slurry) and thin stillage.

### *N. intermedia* pellets fermentation in airlift reactor

The enzymatic hydrolysis of the pretreated slurry (P_201_) was carried out with 7.0% *w*/*v* total solids at pH 5.5 ± 0.1 for 48 h. Hydrolysate slurry was further subjected to centrifugation at 15,000 *g*, and the liquid supernatant was mixed with thin stillage (at 1:1 ratio) to form the fermentation media. The cultivation was carried out in a 4.5 L airlift reactor (Belach Bioteknik, Stockholm, Sweden), with the liquid volume of 3.5 L, for 120 h at 35 °C with an aeration of 1.4 vvm (volume_air_ /volume_media_ /min) following the protocol described in a previous study [19]. The media pH was maintained at 3.5 ± 0.3 throughout the cultivation using 2 M HCl, attributing to the optimum pellet growth condition. *N. intermedia* mycelial pellets, 0.39 ± 0.04 g wet weight (obtained from a pre-culture), was used as the inoculum.

### Inhibitor effect on fungal growth

Cultivations of *N. intermedia* were carried out aerobically in semi synthetic PDB (potato dextrose broth) media containing 20 g/L glucose and 4 g/L potato extract, with varying concentrations (based on the substrate slurry composition) of inhibitors such as., acetic acid (0.5–3.0 g/L), furfural (0.5–2.0 g/L), and hydroxymethylfurfural (HMF, 0.2–5.0 g/L). Batch fermentation in 250 ml Erlenmeyer flasks was carried out for 120 h in a shaking water bath at 35 °C and 150 rpm with samples taken every 24 h. Initial culture pH was adjusted to 3.5 ± 0.1 or 5.5 ± 0.2 with 2 M HCl or 2 M NaOH respectively.

### Analyses

The content of total solids (TS), suspended solids (SS), ash, starch, lignin, and sugars present in the lignocellulosic materials were quantified according to NREL (National Renewable Energy Laboratory) protocols [20–25]. The pH was measured with a digital pH-meter (Philips, PW-9420). Spore concentration was measured using a Bürker counting chamber (with a depth of 0.1 mm) under the light microscope (Carl Zeiss Axiostar plus, Germany). The spore solution was diluted ten times before the measurement, and the spores were counted in a volume of 1/250 μl each. HPLC (Waters 2695, Waters Corporation, USA.) was used to analyze all liquid fractions. A hydrogen-based ion-exchange column (Aminex HPX-87H, Bio-Rad Hercules, CA, U.S.A.) at 60 °C with a Micro-Guard cation-H guard column (Bio-Rad) and 0.6 mL/min 5 mM H_2_SO_4_, used as eluent, was used for the analyses of glucose, ethanol, glycerol and acetic acid, furfural, and 5-hydroxymethyl-furfural. For the separation of glucose, mannose, galactose, cellobiose, xylose, and arabinose, a lead (II) based column (Aminex HPX-87P, Bio-Rad) with two Micro-Guard Deashing (Bio-Rad) precolumns operated at 85 °C with 0.6 mL/min ultrapure water as eluent. Fungal biomass concentration (dry weight) was determined at the end of the cultivation by washing the pellet or mycelial biomass with deionized water followed by drying at 70 °C for 24 h before weight analysis. Limit™ digital Vernier caliper (resolution 0.01 mm) was used to measure the pellet size (diameter). Fermentation media viscosity was measured using a Brookfield digital viscometer-model DV-E (Chemical Instruments AB, Sweden).

All the results and values represented were the average of two independent experimental runs and reported intervals and error bars are ±2 standard deviations, unless otherwise specified. All the data were considered statistically significant at the 95% confidence interval with the *P* value < 0.05.

### Rheological study

The rheological aspects of the *N. intermedia* fermentation in the straw hydrolysate media were analyzed based on the existing concept of the microbial fermentation process. It is estimated that the resistance to flow or viscosity in a fermentation media could either be constant or change during the fermentation process. The relationship between the shear stress τ (Pa) and the shear rate $\frac{dv}{dy}$ or ý (s^− 1^) when the viscosity is constant is given by Newton’s law as shown in eq. 1.

1 $$ \tau =-\upmu \frac{\mathrm{dv}}{\mathrm{dy}}=-\upmu \acute{\mathrm{y}}, $$

However, when the microorganism in the fermentation media grows up to an extent when the viscosity of the media is no longer constant, the fluid becomes non-Newtonian. The flow under this condition is pseudoplastic and it follows the relationship shown in eq. 2, where *K* is the fluid consistency index (Pa.s^n^) and n is a number less than one [26].

2 $$ \tau =\mathrm{K}{\acute{\mathrm{y}}}^n, $$

The viscosity under non-Newtonian condition is the apparent viscosity (μ_a_) and can be expressed by the relationship shown in eq. 3.

3 $$ {\upmu}_{\mathrm{a}}=\mathrm{K}{\acute{\mathrm{y}}}^{\mathrm{n}-1}=\frac{\tau }{\acute{\mathrm{y}}}, $$

## Results

### *N. intermedia* in the integrated ethanol process

#### Effect of lignocellulose pretreatment on fermentation

In order to facilitate the integrated model of first and second-generation ethanol processes, a specially designed fermentation media composed of dilute acid pretreated wheat straw hydrolysate and thin stillage, was used for the filamentous fungal cultivations (Section “Neurospora intermedia fermentation for the integration process”). From all the three hydrolysates used (H_190,_ H_195,_ and H_201_), the slurry obtained from the pretreatment at conditions 0.7% (*w*/*v*) acid conc. at 201 ± 4 °C for 7 min (i.e. H_201_), showed the highest ethanol and fungal biomass production in all the cultivations, both individually and in combination with thin stillage (Tables 2, 3 and 4). With the integration of thin stillage and with the inoculation using pellets, 0.188 ± 0.005 g ethanol /g dry substrate was obtained from enzymatically hydrolyzed straw slurry- H_201_ (3.5% total solids), with a reduced fermentation period to 24 h. However, the cultivation using only the hydrolysate slurry, resulted in an ethanol production as low as only 0.01 ± 0.08 g/g dry substrate straw. Similar results were also obtained with clear liquid hydrolysate (Table 2). With the filamentous mycelial forms as inoculum and with the integration of thin stillage to liquid hydrolysate, the ethanol production was increased from 0.054 ± 0.002 g/g substrate to about 0.23 ± 0.05 g/g dry substrate straw (Table 2) using enzymatically hydrolyzed straw slurry- H_201_ (7% total solids). The overall fermentation results suggest that while using the hydrolysate from different pretreatment conditions (H_201_, H_195_, H_190_), an improved fermentation process with high ethanol and fungal biomass production was observed only with the integrated media using thin stillage (Tables 2, 3 and 4). An improved growth of fungus in the mild pretreated wheat straw slurry (H_201_), where only 0.7% (*w*/*v*) acid concentration has been used, indicated the strong influence of acid loading on the subsequent fungal fermentation process. However, the effect of the dilute acid pretreatment on the ethanol fermentation was considerably reduced when using the integrated media using thin stillage. At various cultivation conditions using substrate hydrolysate with 3.5% total solids, an increase in ethanol yield by about 345, 394 and 544% for the liquid hydrolysate and 3561, 2293%_;_ and 4213% for the slurry (solid and liquid) hydrolysate of H_201,_ H_195_, and H_190_ respectively, was obtained while integrating thin stillage as a nutrient supplement for the fermentation media (Tables 2, 3 and 4).

**Table 2:** Fermentation profile by *N. intermedia* on integrated fermentation substrate using wheat straw pretreated with dilute phosphoric acid (H_201_- acid concentration of 0.7% (*w*/*v*), duration of 7 min, and temperature of 201 ± 4 °C) and thin stillage (T_H_S) mixture

Substrate combination (% *w*/*v* pretreated wheat straw + thin stillage)	Inoculation mode	Mycelial growth form	Dry Biomass (g/L)	Ethanol max (g/L)	Ethanol max- Fermentation time (h)
*7% solid loading*					
WS_S_ + T_H_S	Pellets	Filamentous	16.84 ± 0.25	7.76 ± 0.01	48
	Filamentous	Filamentous	18.61 ± 0.2	9.73 ± 0.3	48
WS_S_	Pellets	–	**–**	**–**	–
	Filamentous	Filamentous	19.27 ± 0.15	0.32 ± 0.15	48
WS_L_+ T_H_S	Pellets	Filamentous	10.78 ± 0.12	10.28 ± 0.1	48
	Filamentous	Filamentous	11.19 ± 0.3	10.06 ± 0.6	48
WS_L_	Pellets	Pellet	3.84 ± 0.12	0.18 ± 0.05	72
	Filamentous	Filamentous	3.44 ± 0.1	0.44 ± 0.10	72
*3.5% solid loading*					
WS_s_ + T_H_S	Pellets	Loose Filamentous	14.01 ± 0.2	6.59 ± 0.1	24
	Filamentous	Filamentous	8.52 ± 0.1	6.9 ± 0.3	24
WS_s_	Pellets	Pellets	16.14 ± 0.3	0.18 ± 0.02	72
	Filamentous	Filamentous	15.63 ± 0.2	0.07 ± 0.02	48
WS_L_+ T_H_S	Pellets	Loose Filamentous	10.46 ± 0.15	7.35 ± 0.10	48
	Filamentous	Filamentous	9.7 ± 0.2	8.34 ± 0.15	48
WS_L_	Pellets	Pellets	3.52 ± 0.1	0.49 ± 0.01	48
	Filamentous	Filamentous	2.64 ± 0.1	1.87 ± 0.04	48

WS_S_, wheat straw slurry; WS_L_, wheat straw liquid hydrolysate after enzymatic hydrolysis

**Table 3:** Fermentation profile by *N. intermedia* on integrated fermentation substrate using wheat straw pretreated with dilute phosphoric acid (H_195_- acid concentration of 1.2% (w/v), duration of 7 min, and temperature of 195 ± 2 °C) and thin stillage (T_H_S) mixture

Substrate combination (%w/v pretreated wheat straw + thin stillage)	Inoculation mode	Mycelial growth form	Dry Biomass (g/L)	Ethanol max (g/L)	Ethanol max- Fermentation time (h)
*7% solid loading*					
WS_S_ + T_H_S	Pellets	Filamentous	21.49 ± 1.18	7.18 ± 0.87	72
	Filamentous	Filamentous	17.78 ± 0.47	8.04 ± 0.07	48
WS_S_	Pellets	–	**–**	**–**	–
	Filamentous	–	–	–	–
WS_L_+ T_H_S	Pellets	Filamentous	13.61 ± 1.08	8.45 ± 0.77	48
	Filamentous	Filamentous	6.62 ± 0.24	4.52 ± 0.78	48
7% WS_L_	Pellets	–	–	–	–
	Filamentous	–	–	–	–
*3.5% solid loading*					
WS_s_ + T_H_S	Pellets	Filamentous	13. 88 ± 0.82	7.89 ± 0.71	48
	Filamentous	Filamentous	8.52 ± 0.62	7.42 ± 0.11	48
WS_s_	Pellets	Pellets	16.84 ± 1.28	0.13 ± 0.08	24
	Filamentous	Filamentous	20.48 ± 1.12	0.31 ± 0.04	48
WS_L_+ T_H_S	Pellets	Filamentous	8.94 ± 0.71	8.35 ± 1.01	48
	Filamentous	Filamentous	13.61 ± 1.02	7.79 ± 0.91	48
WS_L_	Pellets	Pellets	4.73 ± 0.18	1.69 ± 0.87	48
	Filamentous	Filamentous	11.2 ± 0.45	0.13 ± 0.02	24

WS_S_, wheat straw slurry; WS_L_, wheat straw liquid hydrolysate after enzymatic hydrolysis

**Table 4:** Fermentation profile by *N. intermedia* on integrated fermentation substrate using wheat straw pretreated with dilute phosphoric acid (H_190_- acid concentration of 1.75% (w/v), duration of 10 min, and temperature of 190 ± 2 °C) and thin stillage (T_H_S) mixture

Substrate combination (%w/v pretreated wheat straw + thin stillage)	Inoculation mode	Mycelial growth form	Dry Biomass (g/L)	Ethanol max (g/L)	Ethanol max- Fermentation time (h)
*7% solid loading*					
WS_S_ + T_H_S	Pellets	–	–	–	–
	Filamentous	Filamentous	7.88 ± 1.28	6.25 ± 0.77	72
WS_S_	Pellets	–	**–**	**–**	–
	Filamentous	–	–	–	–
WS_L_+ T_H_S	Pellets	–	–	–	–
	Filamentous	Filamentous	6.25 ± 0.18	9.22 ± 1.02	72
WS_L_	Pellets	–	–	–	–
	Filamentous	–	–	–	–
*3.5% solid loading*					
WS_s_ + T_H_S	Pellets	Filamentous	9.22 ± 0.57	6.18 ± 0.85	72
	Filamentous	Filamentous	6.87 ± 0.19	5.54 ± 0.15	72
WS_s_	Pellets	–	–	–	–
	Filamentous	–	–	–	–
WS_L_+ T_H_S	Pellets	Filamentous	7.37 ± 0.88	5.96 ± 0.51	72
	Filamentous	Filamentous	10.95 ± 1.10	6.47 ± 0.79	72
WS_L_	Pellets	Pellets	3.91 ± 0.11	0.11 ± 0.00	48
	Filamentous	Filamentous	3.01 ± 0.07	0.15 ± 0.04	72

WS_S_, wheat straw slurry; WS_L_, wheat straw liquid hydrolysate after enzymatic hydrolysis

*N. intermedia* growth in the form of pellets achieved in the liquid wheat straw hydrolysate showed improved ethanol and inhibitor tolerance (Tables 2, 3, 4 and 5). Fermentation using *N. intermedia* pellets in the liquid straw hydrolysate (WS_L_) at varying substrates solid loading (7 and 3.5%) resulted in up-to 31% increase in the ethanol yield, with an improved glucose assimilation by the pellets (up-to 82% reduction in initial glucose) as opposed to filamentous forms (up-to 51% reduction in initial glucose), under similar culture conditions (Table 2). Considering the ethanol productivity, the concentration was always higher in the integrated media than only the hydrolyzed media. A possible explanation for this fact is the presence of sufficient nutrients in thin stillage needed for the fungi to produce ethanol [27]. Though the fungal biomass concentration was higher in fermentation slurry (substrate hydrolysate) containing higher initial total solids especially for hydrolysate H_201_ (Table 2), the trend was not observed for hydrolysates H_195_, and H_190_. This could be attributed to the presence of higher amount of fermentation inhibitors, especially acetic acid at higher solid loading conditions (Table 1). Similar observations on the production of fungal biomass and ethanol using acid-pretreated wheat straw slurry were obtained in a previous study while using *zygomycetes* strains of filamentous fungus, *Rhizopus* sp.; however the biomass yields obtained was only up to 0.34 g biomass/g consumed monomeric sugars and acetic acid [28].

**Table 5:** Fermentation profile of *N. intermedia* in the presence of inhibitors. Mycelial growth in semi-synthetic potato dextrose media at pH 3.5 represents pellets and at pH 5.5 represents filamentous forms, at varying inhibitor concentrations as observed in wheat straw-hydrolysate at different cultivation conditions

Inhibitor	Concentration in media (g/L)	Mycelial growth form	Dry Biomass (g/L)	Ethanol max (g/L)	Ethanol max- Fermentation time (h)
Furfural	0.5	Pellets	2.57 ± 0.41	2.17 ± 0.78	48
		Filamentous	2.91 ± 0.28	2.73 ± 0.13	72
	1.0	Pellets	2.23 ± 0.11	1.96 ± 0.51	72
		Filamentous	2.09 ± 0.84	1.19 ± 0.01	72
Acetic acid	0.5	Pellets	6.04 ± 0.12	3.17 ± 0.05	48
		Filamentous	7.14 ± 0.98	3.26 ± 0.56	48
	1	Pellets	5.38 ± 0.11	2.14 ± 0.26	72
		Filamentous	6.05 ± 0.25	3.31 ± 0.13	48
	2.0	Pellets	2.52 ± 0.13	1.28 ± 0.02	72
		Filamentous	1.14 ± 0.02	0.87 ± 0.07	72
	3.0	Pellets	0.87 ± 0.08	0.08 ± 0.00	96
		Loose Filamentous	1.05 ± 0.11	–	–
HMF	0.2	Pellets	5.24 ± 0.02	3.97 ± 0.15	48
		Filamentous	6.74 ± 1.01	2.54 ± 0.82	48
	0.5	Pellets	4.81 ± 0.74	3.44 ± 0.43	48
		Filamentous	5.95 ± 0.55	3.31 ± 0.23	48
	1	Pellets	4.52 ± 0.13	3.66 ± 0.42	72
		Filamentous	5.88 ± 0.72	4.30 ± 0.10	48
	3	Pellets	3.26 ± 0.15	2.05 ± 0.48	72
		Filamentous	1.27 ± 0.02	5.51 ± 0.25	48
	5	Pellets	3.52 ± 0.1	1.08 ± 0.00	72
		–	–	–	–

#### Effect of inhibitors and acetic acid assimilation

The growth of fungal pellets in presence of acetic acid, HMF and furfural inhibitors (in liquid semi-synthetic media), at different concentrations, had resulted in increase in the ethanol production by as low as 11% to as high as 45%, compared to filamentous forms at similar growth conditions (Table 5). In this study, considering the relative inhibitor concentrations as represented in the initial slurry or liquid hydrolysate, the major detrimental effect on the fungal growth was observed with acetic acid as compared to other inhibitors (Table 5). The effect of acetic acid inhibition was however reduced by maintaining it in its dissociated form, unavailable for cell-membrane diffusion [29, 30]. This was achieved by a custom-made neutralization step where the extracellular media pH was increased to pH above 8.0 ± 0.5 (almost double the pKa value of acetic acid) using CaCl_2_ (100 mM) and then decreasing the pH to 3.5 ± 0.3 or 5.5 ± 0.2, using 1 M HCl, prior to pellets or filamentous inoculum, respectively. The results showed improved acetic acid assimilation by the fungal cells, with the decrease in its concentration by about 36 to 48%. However, the fungal biomass and ethanol yields decreased considerably with the increase in acetic acid concentration as compared to other fermentation inhibitors (Table 5). The presence of acetic acid in the fermentation media generally leads to a significant decrease in the maximum cell biomass concentration in most cultivations [30, 31].

### Rheological aspects of *N. intermedia* growth and scale-up

**Fig. 2:**
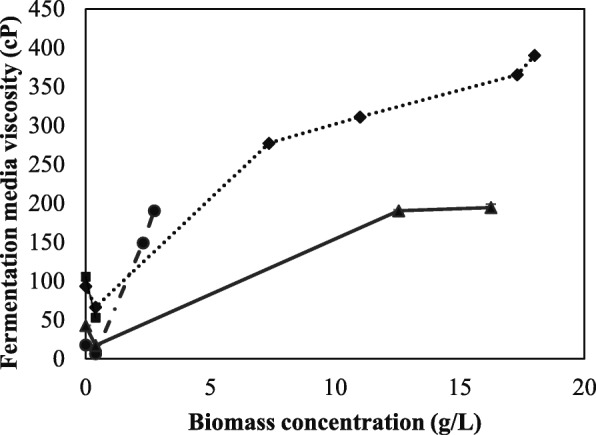
The rheological effect of fermentation media on edible fungal biomass growth pattern. Figure represents wheat straw slurry hydrolysate (H_201_) - at solid hydrolysate loading of 7% hydrolysate (−▲−); at 1:1 mixture of 7% total solid hydrolysate and thin stillage (··■··); at 1:1 mixture of 7% total solid hydrolysate and thin stillage mixture with half initial volume (··♦··), and at clear hydrolysate of 7% solid loading (·−●−·); with each marker points representing 24 h of fermentation period.

The nature of the relationship between the shear stress and the shear rate in a fermentation media determines whether the media would be described as Newtonian or non-Newtonian (eqs. 1 and 2). Most non-viscous fermentation media is usually Newtonian at the beginning of the fermentation process, with the media viscosity being constant until the concentration of the biomass exceeds a threshold value. However, the viscosity would vary considerably, with the increase in the fungal biomass concentration [26]. In general, the viscosity influences oxygen transfer rate into the fermentation medium, which in turn influences the fungal growth. The initial media viscosity was measured to be 89.2 ± 0.7 cP, 102.1 ± 0.4 cP, and 98.3 ± 0.9 cP for the hydrolysate slurry H_201,_ H_195,_ and H_190,_ respectively. When hydrolyzed second generation (wheat straw) substrate H_201_ was combined with first generation substrate (thin stillage), the viscosity of the combination would depend on how the substrates are mixed, together with the total solid content of the media. Fig. 2 represents the effect of viscosity of the fermentation media (such as wheat straw slurry hydrolysate (H_201_); 1:1 mixture of slurry hydrolysate (H_201_) and thin stillage; and the clear liquid supernatant of hydrolysate H_201_)_,_ on the fungal biomass concentration. It was observed that the viscosity of the fermentation media played a critical role in the fungal growth [32], where higher the viscosity, the less the oxygen transfer with reduced biomass yield. Fermentation experiments carried out using 7% (*w*/*v*) hydrolysate H_195_ and H_190_ in different media combinations of the integrated media hence showed no fungal growth attributing to its high viscosity (Tables 3 and 4). However, when the initial cultivation volume of the fermentation media was reduced by half, improved fungal growth was observed until 120 h of fermentation, possibly due to the increased oxygen transfer into the media [33, 34]. The integrated media using 1:1 mixture of 7% *w*/*v* (total solid) slurry hydrolysate (H_201_) and thin stillage with half initial volume, had resulted in a maximum of 18.0 ± 2.8 g/L of fungal biomass, pointing out the obligate aerobic nature of *N. intermedia*. This hence implies the significance of reduced media viscosity and adequate oxygen transfer into bioreactors, for an efficient fermentation using *N. intermedia* in the integrated first and second generation ethanol production process model.

Scale-up of the fermentation experiments using the optimum cultivation media showing the maximum fungal growth and ethanol production was carried out in a 4.5 L bench scale airlift reactor at an aeration rate of 1.4 vvm (as described in section “N. intermedia pellets fermentation in airlift reactor”). Integrated media containing 1:1 mixture of thin stillage and 7% w/v slurry hydrolysate (H_201_) was fed to the airlift reactor in batches. The media viscosity decreased during the stationary phase (24 h fermentation) to 28.1 ± 2.1 cP from the initial 90.2 ± 3.8 cP, which was similar to what was observed for the shake flask experiment (Fig. 2). However, the viscosity of the media increased to 209.8 ± 3.7 cP during the growth phase (48 h cultivations) and then decreased to 180.7 ± 1.8 cP during the stationary phase (72 h cultivations). Cultivations in the airlift under this condition resulted in the production of 18.2 ± 1.3 g/L of fungal biomass (72 h cultivations). However, operating the airlift reactor at 1.4 vvm aeration rates caused foaming and evaporation of the fermentation medium, which was controlled by applying intermitted mixing strategies.

#### Fungal growth morphology and product formation

The previous study has shown that the fungal growth morphology significantly influences the product formation rate, with pellet morphology favoring more ethanol production and filamentous growth favoring more biomass formation [19]. Additionally, different fungi growth morphologies have their advantages and drawbacks from an overall process perspective [35]. However, the oxygen uptake rate for fungi growing as pellets was much higher than that of the filamentous form, which implies that better aeration efficiency is obtained during fungi pellet growth [19]. The results from the fermentation experiments using either pellets or filamentous biomass as inoculum had indicated that the biomass growth could not occur as pellets in the integrated fermentation media (1:1 mixture of thin stillage and straw hydrolysate) in all the cultivation conditions using different substrates hydrolysates (Tables 2, 3 and 4). A possible reason for this is the high viscosity of the media (between 35 and 100 cP), which in turn leads to less oxygen transfer into the fermentation media [36]. Hence, filamentous morphology was the most common biomass form for the integrated media using the hydrolysate with 7% total solid content. Nevertheless, considering biomass growth as pellets (as found in liquid hydrolysate), it was observed that the viscosity of the fermentation media remains within a constant range, independent of the biomass growth rate (or concentrations) as compared to the growth in form of mycelial filaments as found in the fermentation using the integrated media (Fig. 2).

## Discussion

Integrating lignocelluloses (wheat straw) to existing first generation (1G) wheat-based ethanol facilities could possibly reduce its current dependence on the food grains (wheat), in addition to reducing the investment cost and risk associated with the 2G lignocellulose-to-ethanol process [3, 4, 37]. The ascomycetes filamentous fungus *N. intermedia*, capable of utilizing pentoses [2, 38], and traditionally used for the preparation of the indigenous Indonesian food *oncom* [39], was used in the present integration model. The fungal biomass obtained could effectively be used as an animal feed component or enrich the DDGS quality at the 1G-ethanol plants or be considered as a new valuable by-product [11]. However, the practical aspects of fermentation such as fluid rheology, the effect of fermentation inhibitors and the fungal growth morphology, highly affect the biomass growth and ethanol production. This study hence describes for the first time, various process aspects of *N. intermedia* fermentation on wheat-based integrated first and second-generation ethanol substrate. The previous challenges with 2G lignocellulose (wheat straw)-to-ethanol process while using *N. intermedia* [18, 37] could also be effectively addressed with the current integration model. Thin stillage, a process-waste stream at 1G-ethnaol facility, has also been valorized effectively using the integration model. Considering the fermentation inhibitors, the presence of fermentation inhibitors (mainly HMF, furfural and acetic acid) from the pretreatment process had posed severe challenges in *N. intermedia* growth during previous studies [18, 37]. Hence, in this study, the addition of fungal pellets capable of an improved fermentation and inhibitor tolerance [17, 19] was used as the starting inoculum for the cultivation.

### Ethanol and biomass optimization for the integrated process

Higher ethanol and fungal biomass production are always beneficial for increasing the profitability of the integrated process model at the ethanol industries. The current results suggest that the feedstock (from both first and second-generation process) integration model greatly influences the optimal ethanol and biomass production. Integration after the solid removal from the lignocellulose hydrolysate (Fig. 1 b) would result in higher ethanol production as seen in the case of integrated thin stillage and clear hydrolysate (for example H_201_) media as compared to that of the integrated whole slurry hydrolysate and thin stillage media (Tables 2, 3 and 4)_._ However, higher biomass production was most favored while using the whole slurry of the lignocellulose hydrolysate (Fig. 1 a) for the integrated fermentation media (Table 2, 3 and 4). The use of lower solid loading also facilitates the removal of suspended particles from the slurry after enzyme hydrolysis, allowing an easy separation of the fungal biomass. Hence, a trade-off between the fungal biomass and ethanol production clearly exists in the integration model, which would greatly depend on the prevailing market conditions. Nevertheless, from a process standpoint, the integration before solid removal is beneficial for minimizing the associated energy and investment /operation cost associated with the process steps, for example, centrifugation. This could also minimize the investment cost for an ethanol facility, considering that for a typical 100,000 m^3^ ethanol facility, the centrifuge accounts for 18% of the total fermentation investment cost [40]. However, a thorough techno-economic analysis is required to optimize the actual integration model for further developments at a larger scale.

## Conclusions

The use of integrated media with wheat straw (dilute acid pretreated and hydrolyzed) and thin stillage (from first generation ethanol facility), overcomes the challenges previously faced by the filamentous fungi fermentation on wheat straw. The use of *N. intermedia* mycelial pellets as fermentation inoculum resulted in an improved ethanol and inhibitor tolerance, with acetic assimilation by about 36 to 48% (decreasing initial acid concentration). Overall rheological observations coupled with the high biomass yields at lower initial solid loading conditions, points out the significance of adequate oxygen transfer into the bioreactors, as the most critical factor for filamentous fungal growth in the proposed integrated ethanol process.
